# Low Levels of Neutralizing Antibodies Against SARS‐CoV‐2 KP.3.1.1 and XEC in Serum From Seniors in May 2024

**DOI:** 10.1111/irv.70102

**Published:** 2025-05-14

**Authors:** Even Fossum, Elisabeth Lea Vikse, Anna Hayman Robertson, Asia‐Sophia Wolf, Andreas Rohringer, Lill Trogstad, Siri Mjaaland, Olav Hungnes, Karoline Bragstad

**Affiliations:** ^1^ Department of Virology, Division of Infection Control Norwegian Institute of Public Health Oslo Norway; ^2^ Department of Method Development and Analytics, Division of Infection Control Norwegian Institute of Public Health Oslo Norway

**Keywords:** elderly, immunity, KP.3.1.1, neutralizing antibodies, SARS‐CoV‐2, XEC

## Abstract

New immune evasive variants of SARS‐CoV‐2 may increase infections and hospitalizations in risk groups, such as the elderly. In this study, we evaluated neutralizing antibodies against KP.3.1.1 and XEC, virus variants that were either widely distributed or on the rise globally in the fall of 2024, in sera from a cohort of seniors aged 68–82 years collected in April/May 2024. Neutralizing responses were low against both KP.3.1.1 and XEC, also in XBB.1.5 boosted individuals and people with recent break‐through infections, supporting the recommendation of an updated COVID‐19 vaccine booster in this age group.

## 1 Introduction

**FIGURE 1:**
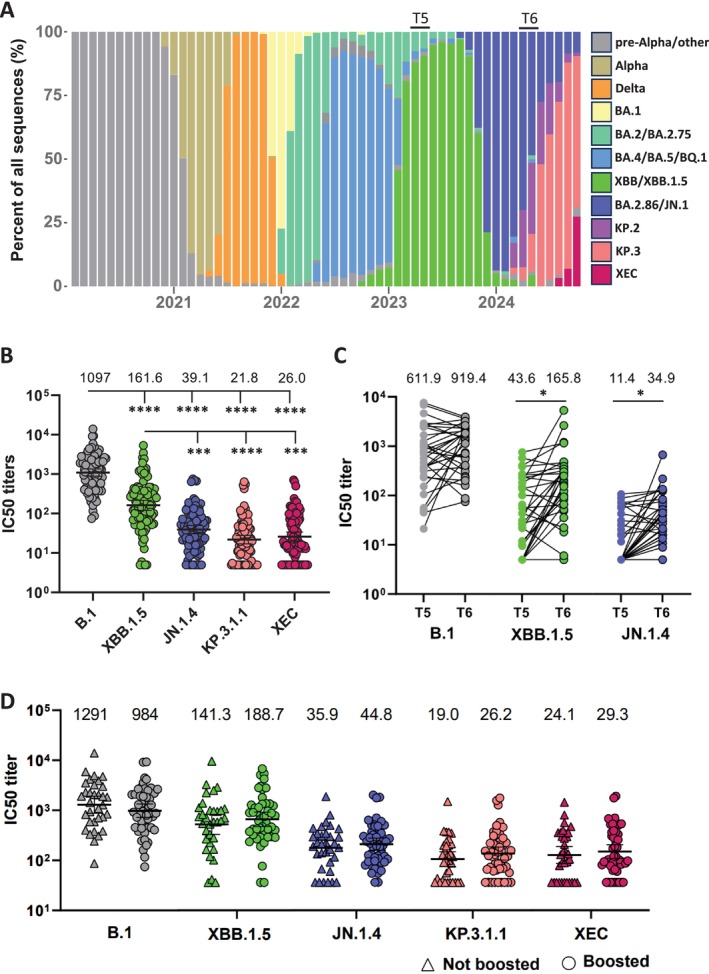
Neutralizing titers against SARS‐CoV‐2 variants in sera from seniors. (A) Prevalence of SARS‐CoV‐2 variants in Norway determined by whole‐genome sequencing from January 2020 to October 2024. T5 and T6 indicate time points for serum sampling in April/May 2023 and April/May 2024, respectively. (B) Neutralizing titers in seniors (*n* = 97) against B.1, XBB.1.5, JN.1.4, KP.3.1.1 and XEC in serum samples collected in April/May 2024. The number above each column indicates geometric mean titer. (C) Change in neutralizing titers against B.1, XBB.1.5 and JN.1.4 in 33 individuals who donated sera at time points T5 and T6. (D) Serum samples from April/May 2024 were divided into two groups depending on whether the donor reported to have received the monovalent XBB.1.5 vaccine, excluding individuals with reported infection between time points T5 and T6. In total, 48 donors had received an XBB.1.5 booster dose and 33 donors had not. The number above each column indicates geometric mean titer. The data presented in panels (B)–(D) are IC50 neutralizing titers, with geometric mean ± 95% CI shown in panels (B) and (D). Significant difference analyzed in (B) by one‐way ANOVA with Tukey's multiple comparison test, in (C) by paired *t*‐test and in (D) by Mann–Whitney test. *p < 0.05, ***p < 0.001, and ****p < 0.0001.

Vaccination and infections have contributed to a high level of immunity against SARS‐CoV‐2 in the Norwegian population [1]. Nevertheless, new immune evasive SARS‐CoV‐2 variants continue to emerge and cause periodic increases in infections and hospitalizations, especially in risk groups such as the elderly [2]. August 2023 saw the emergence of the BA.2.86 variant that differed from the then circulating XBB–derived EG.5.1 strain by > 30 mutations in the Spike protein [3]. In Norway, BA.2.86–derived JN.1 variants became dominant in October/November 2023 (Figure 1A). Infections with the JN.1–derived KP.2 variants, which were defined by the addition of the so‐called “FLiRT” mutations (F456L and R346T), started to increase from March 2024 [4]. KP.2 variants were from June 2024 overtaken by KP.3 variants containing the “FLuQE” mutations (F456L and Q493E), and since July, the KP.3.1.1 variant containing the S31 deletion has dominated [5]. Since August 2024, the prevalence of the XEC‐variant (recombination of BA.2.86‐derived variants KS.1 and KP.3.3) has started to rise, with recent studies suggesting greater immune evasion compared with KP.3.1.1 [6]. To evaluate the existing humoral immunity in the elderly prior to the winter season 2024/2025, we performed neutralization assays against KP.3.1.1 and XEC on serum samples harvested from seniors in April/May 2024.

## 2 Method

The Senior Cohort was established towards the end of 2020 to study the consequences of COVID‐19 disease and the effects and safety of COVID‐19 vaccines in elderly individuals. Approximately 5000 individuals aged 65–80 years living in Oslo, Norway, consented to participate in answering regular questionnaires, and approximately 500 individuals consented to donating longitudinal blood samples, as previously described [7]. Of these, 97 individuals donated blood in April/May 2024. Information on individual vaccination and infection status was obtained through regular questionnaires from May 2021 up to the time of serum donation, in addition through the Norwegian Immunization Registry (SYSVAK) and the Norwegian Surveillance System for Communicable Disease (MSIS). Information on the XBB.1.5 booster vaccine administered in the fall of 2023 was obtained through self‐reporting and limited to the month of vaccination.

SARS‐CoV‐2 variants were isolated from clinical samples submitted for surveillance to the Norwegian Institute of Public Health (NIPH) in VeroE6‐TMPRSS2 cells as previously described [8]. JN.1.4 (available in GISAID EpiCoV with accession number EPI_ISL_18735198), KP.3.1.1 (EPI_ISL_19268292), and XEC (EPI_ISL_19393351) were passaged twice in VeroE6 before titration and assessed in a live virus neutralization assay as previously described [8]. SARS‐CoV‐2 variants B.1 (EPI_ISL_449791) and XBB.1.5 (EPI_ISL_16969674) were previously isolated [1].

## 3 Reduced Neutralizing Titers Against KP.3.1.1 and XEC

To evaluate immunity in seniors in Norway against recent SARS‐CoV‐2 variants, 97 serum samples were harvested from individuals aged 68–82 years in April/May 2024. At the time, BA.2.86–derived JN.1 variants were dominant in Norway, although infections with BA.2.86–derived KP.2 and KP.3 variants were increasing (Figure 1A). All individuals had received at least three doses of mRNA vaccine (range three to six doses with a median of five doses), resulting in 37 different combinations of vaccines from Pfizer and/or Moderna. In addition, 59 (60.8%) individuals had reported at least one previous breakthrough infections, thus reflecting the heterogeneity of immunity currently present in Norwegian seniors. The serum samples were analyzed in a live‐virus neutralization assay against SARS‐CoV‐2 variants B.1, XBB.1.5, JN.1.4, KP.3.1.1, and XEC (Figure 1B).

While neutralizing titers against B.1 were high, there was a significant reduction in neutralization of XBB.1.5, JN.1.4, KP.3.1.1, and XEC. Neutralizing titers against XBB.1.5 were also significantly higher than JN.1.4, KP.3.1.1, and XEC (Figure 1B). Neutralizing titers against JN.1.4 were higher than KP.3.1.1 and XEC, but the difference was not significant (Figure 1B). For KP.3.1.1 and XEC, the levels of neutralizing titers were very low. We did not see a further reduction for XEC compared with KP.3.1.1 as has been reported in pseudo‐neutralization assays [6].

Of the 97 individuals that donated serum in April/May 2024 (“T6”), 33 individuals had also donated sera a year earlier in April/May 2023 (“T5”). Interestingly, the level of neutralization for B.1 remained constant from 1 year to the next (Figure 1C), which could reflect continuous back‐boosting of B.1 neutralizing responses through infections and/or vaccination. For both XBB.1.5 and JN.1.4, there was a significant increase in neutralizing antibodies from 2023 to 2024 potentially reflecting administration of the monovalent XBB.1.5 vaccine in the fall of 2023 and circulation of the XBB.1.5‐derived variants throughout 2023 and JN.1‐derived variants from November 2023 (Figure 1A,C).

To assess if the XBB.1.5 booster vaccine administered in from September 2023 influenced neutralizing antibody responses in serum samples collected in April/May 2024, serum samples were divided into boosted and un‐boosted based on XBB.1.5 vaccination. Individuals that had reported a positive SARS‐CoV‐2 test between time points T5 and T6 (*n* = 16) were excluded from the analysis. While boosted individuals did have higher neutralizing titers against XBB.1.5, JN.1.4, KP.3.1.1, and XEC, the difference was not significant for any of the variants (Figure 1D). There was a tendency for decreased NAb against newer SARS‐CoV‐2 variants for donors who had a Wuhan‐based mRNA vaccine as their last vaccine dose, but the difference was not significant compared to donors with an XBB.1.5 or a bivalent vaccine as their last dose (Figure S1).

We did not observe any significant differences between individuals with a recently reported SARS‐CoV‐2 infection between T5 and T6 compared with those without when XBB.1.5 boosted individuals were removed from the analysis (Figure S2A). However, we observed that 4/9 individuals without reported infection or XBB.1.5 vaccination had a > 5‐fold increase in NAb titers against JN.1.4 from T5 to T6. The results may reflect mild or asymptomatic infections resulting in underreporting of the true number of infections and masking of any differences between infected and non‐infected (Figure S2B).

## 4 Discussion

Serum levels of neutralizing antibodies (NAb) are recognized as a correlate of protection against SARS‐CoV‐2 infection after vaccination [9, 10], although it is less clear what constitutes a protective level of NAb. Immunity other than NAb also contribute to protection, especially after infection [11]. The significant reduction in neutralizing antibody titer from B.1 and XBB.1.5 to KP.3.1.1 and XEC does, however, indicate that the seniors had reduced protection against infection with current strains in the summer of 2024. Our results also indicate that prior immunization with the XBB.1.5 monovalent booster in the fall of 2023 was unlikely to protect against infection with the KP.3.1.1 and XEC strains in the fall/winter of 2024, which is in accordance with a study showing limited protection from infection > 12 weeks after administration of the XBB.1.5 vaccine [12]. However, immunization with an updated JN.1 or KP.2 monovalent vaccine have been reported to increase neutralizing antibodies against the KP.3.1.1 and XEC variants [13]. Interestingly, a recent study from Denmark, Finland and Sweden indicates a 57.9% protection from hospitalization and 75.2% protection from death in persons over 65 years over a 24 weeks follow‐up after XBB.1.5 booster vaccination [14]. A concern is that only 54% of individuals in Norway aged 65 years or older took a SARS‐CoV‐2 booster vaccine during the 2023/2024 season [15], highlighting a need to better inform this age group of the benefits of boosting immune responses with an updated SARS‐CoV‐2 vaccine.

A limitation of our study is that data on vaccinations from September 2023 and on infections from January 2022 were obtained through self‐reporting, and we can therefore not rule out errors or underreporting. However, previous data from the same individuals on self‐reported immunization and infection have been highly consistent with data from the health registries (SYSVAK and MSIS). Nevertheless, unreported subclinical or mild infections may have influenced the comparison of XBB.1.5 boosted vs. un‐boosted individuals, as well as recently infected vs non‐infected. Indeed, Figure S2B indicates that 44% individuals without reported infection or vaccination had an increase in NAb against JN.1.4 at T6, compared with T5, potentially reflecting an unreported infection. It should also be noted that our study only evaluates neutralizing antibodies against recent strains and does not consider the role of T cells. Indeed, previous studies with the same cohort have indicated that T cell responses are more stable against new virus variants [7] and also contribute to protection against severe disease [16].

## 5 Conclusion

Our study indicates that seniors had very low levels of neutralizing antibodies against recent SARS‐CoV‐2 strains KP.3.1.1 and XEC in the summer of 2024. Previous vaccination with an XBB.1.5 booster in the fall of 2023 or recent infection did not significantly increase neutralizing antibody titers against strains circulating in the winter of 2024/2025. The results support current recommendation of an updated booster covid‐19 vaccines for this age‐group.

## Author Contributions

**Even Fossum:** writing – original draft, writing – review and editing, investigation, methodology, conceptualization. **Elisabeth Lea Vikse:** writing – review and editing, investigation, methodology, conceptualization. **Anna Hayman Robertson:** writing – review and editing, conceptualization, resources, data curation. **Asia‐Sophia Wolf:** writing – review and editing, conceptualization, resources, data curation. **Andreas Rohringer:** writing – review and editing, conceptualization, data curation, visualization. **Lill Trogstad:** writing – review and editing, conceptualization, resources. **Siri Mjaaland:** conceptualization, writing – review and editing, resources. **Olav Hungnes:** conceptualization, supervision, writing – review and editing. **Karoline Bragstad:** conceptualization, supervision, writing – review and editing, project administration.

## Ethics Statement

Blood samples and medical information were collected with written consent from all participants. The study was approved by the Regional Committees for Medical and Health Research Ethics Southeast (REK) with reference nr. 229359. All data were pseudo anonymized to protect patient privacy and confidentiality.

## Conflicts of Interest

The authors declare no conflicts of interest.

### Peer Review

The peer review history for this article is available at https://www.webofscience.com/api/gateway/wos/peer‐review/10.1111/irv.70102.

## Supporting information


**Figure S1.** Neutralizing titers against SARS‐CoV‐2 variants following vaccination. Neutralizing titers against B.1, XBB.1.5, JN.1.4, KP.3.1.1, and XEC in serum samples collected in April/May 2024 divided into groups based on last administered vaccine type. Monovalent XBB.1.5 vaccine was administered < 265 days before serum collection (*n* = 48), bivalent BA.1/Wuhan or BA.5/Wuhan was administered 339–589 days before serum collection (*n* = 22) and monovalent Wuhan based vaccine was administered 594–904 days before serum collection (*n* = 11). Samples with recently reported infection between time points T5 and T6 were exclude from the analysis (*n* = 16). The number above each column indicates geometric mean titer. The data presented are IC50 neutralizing titers, with geometric mean ± 95% CI. Significant differences were analyzed by one‐way ANOVA with Tukey's multiple comparison test.
**Figure S2.** Neutralizing titers against SARS‐CoV‐2 variants following recent infection. (A) Neutralizing titers against B.1, XBB.1.5, JN.1.4, KP.3.1.1, and XEC in serum samples collected in April/May 2024 from donors that have reported a recent infection between time point T5 and T6 (*n* = 12) vs donors that have reported not being infected (*n* = 26). Donors that received an XBB.1.5 booster dose between T5 and T6 (*n* = 52), and donors that did not report infection status between T5 and T6 (*n* = 7) were excluded from the analysis. (B) Neutralizing titers against B.1, XBB.1.5 and JN.1.4 in 9 individuals who donated sera at time points T5 and T6, and who have not reported being infected or vaccinated with the XBB1.5 booster between T5 and T6. (A, B) The number above each column indicates geometric mean titer, and the data presented in (A) are IC50 neutralizing titers, with geometric mean ± 95% CI. Significant differences were analyzed by (A) Mann–Whitney test and (B) paired *t*‐test.

## Data Availability

Anonymized data can be shared in accordance with the data sharing policy of NIPH. Preprint: BioRxiv: https://doi.org/10.1101/2024.11.18.623742.
