# Supplementary material for: Low Levels of Neutralizing Antibodies Against SARS‐CoV‐2 KP.3.1.1 and XEC in Serum From Seniors in May 2024
Source: Influenza Other Respir Viruses. 2025 May 14;19(5):e70102. doi: 10.1111/irv.70102 (PMC12078752; doi:10.1111/irv.70102)

Supplementary information:

**Supplementary Figure 1: Neutralizing titers against SARS-CoV-2 variants following vaccination.** Neutralizing titers against B.1, XBB.1.5, JN.1.4, KP.3.1.1 and XEC in serum samples collected in April/May 2024 divided into groups based on last administered vaccine type. Monovalen XBB.1.5 vaccine was administered < 265 days before serum collection (n = 48), bivalent BA.1/Wuhan or BA.5/Wuhan was administered 339 – 589 days before serum collection (n = 22) and monovalent Wuhan based vaccine was administered 594 – 904 days before serum collection (n = 11). Samples with recently reported infection between time points T5 and T6 were exclude from the analysis (n = 16). The number above each column indicates geometric mean titer. The data presented are IC50 neutralizing titers, with geometric mean ± 95% CI. Significant differences were analyzed by one-way ANOVA with Tukey`s multiple comparison test.

**Supplementary Figure 2: Neutralizing titers against SARS-CoV-2 variants following recent infection. A)** Neutralizing titers against B.1, XBB.1.5, JN.1.4, KP.3.1.1 and XEC in serum samples collected in April/May 2024 from donors that have reported a recent infection between time point T5 and T6 (n = 12) vs donors that have reported not being infected (n = 26). Donors that received an XBB.1.5 boosted dose between T5 and T6 (n = 52), and donors did not report infection status between T5 and T6 (n = 7) were excluded from the analysis. **B)** Neutralizing titers against B.1, XBB.1.5 and JN.1.4 in 9 individuals who donated sera at time points T5 and T6, and who have not reported being infected or vaccinated with the XBB1.5 booster between T5 and T6. **A – B)** The number above each column indicates geometric mean titer, and the data presented in **A)** are IC50 neutralizing titers, with geometric mean ± 95% CI. Significant differences were analyzed by **A)** Mann-Whitney test and **B)** Paired t-test.

# Supplementary Figure 1

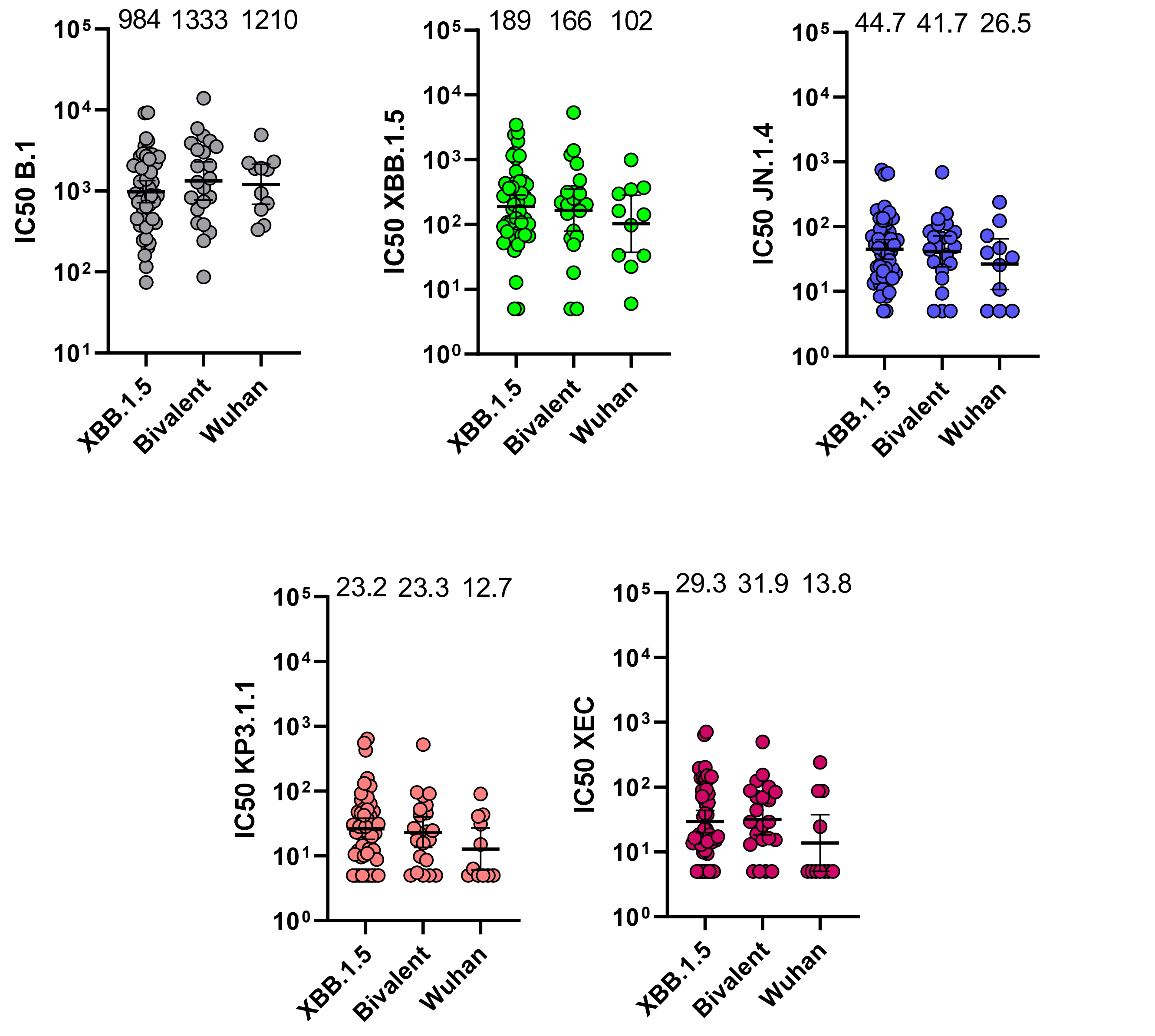

# Supplementary Figure 2

## A

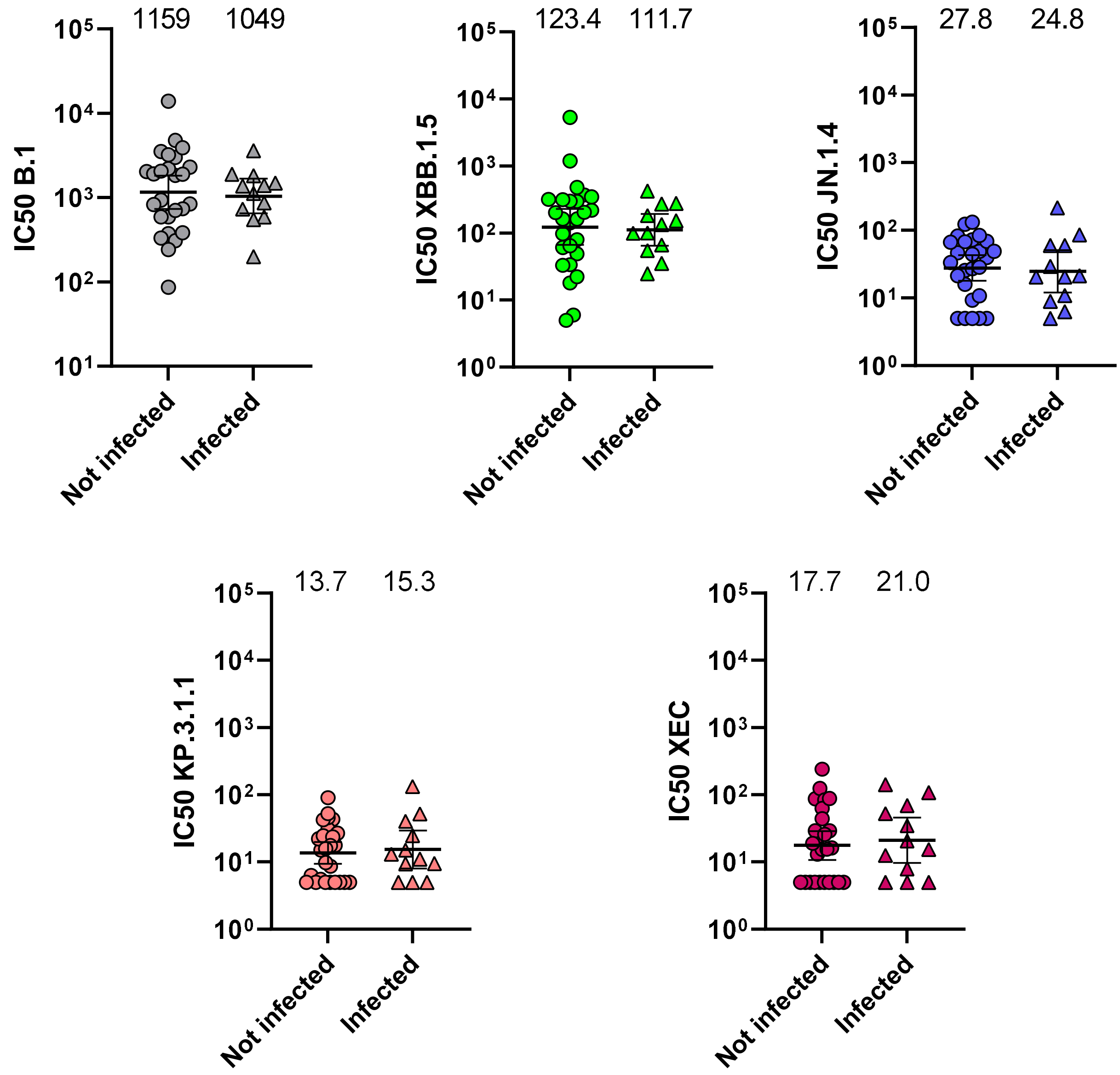


## B

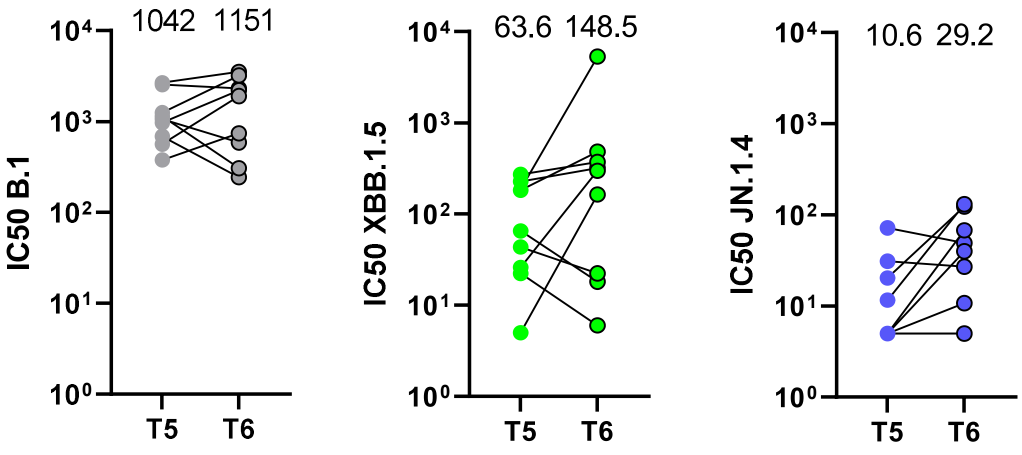

Supplement: Supplementary file 1 — Figure S1. Neutralizing titers against SARS‐CoV‐2 variants following vaccination. Neutralizing titers against B.1, XBB.1.5, JN.1.4, KP.3.1.1, and XEC in serum samples collected in April/May 2024 divided into groups based on last administered vaccine type. Monovalent XBB.1.5 vaccine was administered < 265 days before serum collection (n = 48), bivalent BA.1/Wuhan or BA.5/Wuhan was administered 339–589 days before serum collection (n = 22) and monovalent Wuhan based vaccine was administered 594–904 days before serum collection (n = 11). Samples with recently reported infection between time points T5 and T6 were exclude from the analysis (n = 16). The number above each column indicates geometric mean titer. The data presented are IC50 neutralizing titers, with geometric mean ± 95% CI. Significant differences were analyzed by one‐way ANOVA with Tukey's multiple comparison test. Figure S2. Neutralizing titers against SARS‐CoV‐2 variants following recent infection. (A) Neutralizing titers against B.1, XBB.1.5, JN.1.4, KP.3.1.1, and XEC in serum samples collected in April/May 2024 from donors that have reported a recent infection between time point T5 and T6 (n = 12) vs donors that have reported not being infected (n = 26). Donors that received an XBB.1.5 booster dose between T5 and T6 (n = 52), and donors that did not report infection status between T5 and T6 (n = 7) were excluded from the analysis. (B) Neutralizing titers against B.1, XBB.1.5 and JN.1.4 in 9 individuals who donated sera at time points T5 and T6, and who have not reported being infected or vaccinated with the XBB1.5 booster between T5 and T6. (A, B) The number above each column indicates geometric mean titer, and the data presented in (A) are IC50 neutralizing titers, with geometric mean ± 95% CI. Significant differences were analyzed by (A) Mann–Whitney test and (B) paired t‐test. [file IRV-19-e70102-s001.pdf]
